# Elucidating the direct effects of the novel HDAC inhibitor bocodepsin (OKI-179) on T cells to rationally design regimens for combining with immunotherapy

**DOI:** 10.3389/fimmu.2023.1260545

**Published:** 2023-09-06

**Authors:** Nisha Holay, Alexander Somma, Mark Duchow, Milad Soleimani, Anna Capasso, Srividya Kottapalli, Joshua Rios, Uma Giri, Jennifer Diamond, Anna Schreiber, Anthony D. Piscopio, Carla Van Den Berg, S. Gail Eckhardt, Todd A. Triplett

**Affiliations:** ^1^ Interdisciplinary Life Sciences Graduate Programs, The University of Texas at Austin, Austin, TX, United States; ^2^ Livestrong Cancer Institutes, Department of Oncology, Dell Medical School, The University of Texas at Austin, Austin, TX, United States; ^3^ OnKure Therapeutics, Boulder, CO, United States; ^4^ University of Colorado Cancer Center, University of Colorado Anschutz Medical Campus, Denver, CO, United States; ^5^ Division of Pharmacology and Toxicology, College of Pharmacy, University of Texas at Austin, Austin, TX, United States; ^6^ Department of Immunotherapeutics & Biotechnology, School of Pharmacy, Texas Tech University Health Sciences Center, Abilene, TX, United States

**Keywords:** T cell, colorectal cancer, HDAC, HDAC inhibitors, cancer, immunotherapy, immunology

## Abstract

Histone deacetylase inhibitors (HDACi) are currently being explored for the treatment of both solid and hematological malignancies. Although originally thought to exert cytotoxic responses through tumor-intrinsic mechanisms by increasing expression of tumor suppressor genes, several studies have demonstrated that therapeutic responses depend on an intact adaptive immune system: particularly CD8 T cells. It is therefore critical to understand how HDACi directly affects T cells in order to rationally design regimens for combining with immunotherapy. In this study, we evaluated T cell responses to a novel class-selective HDACi (OKI-179, bocodepsin) by assessing histone acetylation levels, which revealed rapid responsiveness accompanied by an increase in CD4 and CD8 T cell frequencies in the blood. However, these rapid responses were transient, as histone acetylation and frequencies waned within 24 hours. This contrasts with *in vitro* models where high acetylation was sustained and continuous exposure to HDACi suppressed cytokine production. *In vivo* comparisons demonstrated that stopping OKI-179 treatment during PD-1 blockade was superior to continuous treatment. These findings provide novel insight into the direct effects of HDAC inhibitors on T cells and that treatment schedules that take into account acute T cell effects should be considered when combined with immunotherapies in order to fully harness the tumor-specific T cell responses in patients.

## 1 Introduction

Histone deacetylases (HDACs) are a collection of proteins which catalyze the removal of acetyl groups from lysine residues of proteins. While originally recognized for decreasing gene expression by deacetylating histones, thus leading to a closed chromatin state, altered acetylation states and aberrantly overexpressed HDACs have been found in multiple tumor types, ultimately leading to the use of HDAC inhibitors (HDACi) to treat cancer patients (1). Historically, HDACi were postulated to exert cytotoxic effects directly on malignant cells via tumor-intrinsic mechanisms, such as altering gene expression that leads to cell cycle arrest and apoptosis (2). However, more recent analyses have shown that HDACs regulate acetylation of non-histone proteins that highlight the potential of HDACi affecting tumor growth via other mechanisms (2, 3). Importantly, HDACi have also been shown to modulate the immune system (4, 5). Furthermore, multiple recent studies demonstrate that responses to monotherapies with either class-selective and non-selective inhibitors can be immune-mediated, as indicated by the loss of efficacy in the absence of an adaptive immune system (6–8). Additional investigations have further pinpointed T cells as being the ultimate mediators of tumor regression because depletion of CD8 T cells has been shown to abolish the anti-tumor effect in multiple models (6, 9, 10). Furthermore, synergistic effects of HDACi can be obtained when combined with immunotherapies that target T cells (5, 11) such as checkpoint blockade with αPD-1 (5, 11), adoptive cell therapy models (12), and other modalities of immunotherapy (13). Altogether, these findings highlight the potential of combining HDACi with immunotherapy to achieve complete tumor regression through an improved understanding of how inhibitors impact immune responses.

Although the mechanism behind anti-tumor T cell responses induced by HDACi treatment is unclear, HDACi have been reported to affect multiple nodes involved in anti-tumor immune responses. This includes altering expression of tumor antigens (12, 14, 15), upregulating antigen-presenting machinery (MHC-I/II) (16, 17), enhancing secretion of T cell-recruiting chemokines (4), rendering tumors susceptible to death receptor killing (12, 18), and altering the balance of suppressive and inflammatory myeloid cells (19, 20). However, whether part of the mechanism is mediated through direct effects on T cells is unknown as previous analyses have demonstrated HDACs also play various roles in T cell biology. Because T cells have been shown to be the ultimate mediators, understanding how T cells are affected by specific inhibitors is important as it could ultimately help rationally design combination regimens for achieving maximal responses to immunotherapy. While there have been investigations evaluating the effects of HDACi on T cells, these findings often produced contradictory results that may be attributed to differences between *in vitro* or *in vivo* models (21–25). Thus, additional investigations are needed to provide a more comprehensive understanding of how HDACi impact T cells in patients.

Therefore, in this study we integrated results from analyses of T cell responses in patients treated with OKI-179 (bocodepsin) in a phase I dose-escalation study, together with *in vitro* and *in vivo* murine models in order to provide a more comprehensive picture of the direct effects of HDACi on T cells. As previously described, OKI-179 is a pro-drug analog of largazole, which is a natural compound whose structure partially resembles romidepsin (22, 26). However, unlike largazole and romidepsin, OKI-179 is orally bioavailable and has been shown to be effective in eliciting tumor regression when administered orally in multiple murine tumor models (27, 28). Importantly, OKI-179 has also been shown to potentiate responses to immunotherapy with αPD-1 in murine studies using a syngeneic B cell-lymphoma model (16) as well as in humanized mouse models harboring triple-negative breast cancer tumors (29). Given its ability to enhance anti-tumor immune responses, we sought to determine the direct effects on T cells. Analysis of blood samples collected longitudinally, revealed that T cells rapidly respond to OKI-179, as indicated by a sharp increase in histone acetylation levels as well as an increase in frequency among peripheral blood mononuclear cells (PBMCs). However, these responses were transient as both histone acetylation levels and T cell composition changes waned within 24 hours following treatment. This contrasts with *in vitro* assays where high acetylation levels in T cells were maintained over this timeframe due to continuous exposure to high HDACi, led to suppressing effector cytokine functions. Removing the inhibitor *in vitro* to better mimic the rapid responses in patients revealed that these suppressive effects were largely reversible, as cytokine production was restored. Consistent with these effects, our *in vivo* data demonstrate that stopping OKI-179 during immunotherapy resulted in superior anti-tumor responses compared to continuous treatment.

## 2 Materials and methods

### 2.1 Compounds

OKI-005 and OKI-179 were provided by OnKure Therapeutics (Boulder, CO). Due to their different chemical structures described elsewhere (27), OKI-005 dissolved in DMSO was used for all *in vitro* analyses, while OKI-179 was used for all *in vivo* studies in murine models and in patients enrolled in a phase-I clinical trial, dissolved in 0.1 M citric acid. The HDAC inhibitors, entinostat, vorinostat and panobinostat and the SHP2 inhibitor SHP099 were all purchased from Selleck Chemicals and dissolved in DMSO.

### 2.2 Cell culture

Mouse colorectal adenocarcinoma MC38 (Kerafast; RRID: CVCL_B288) cells were cultured in Dulbecco’s Modified Eagle Medium (DMEM) (Gibco). Healthy donor human peripheral blood mononuclear cells (PBMCs) were cultured in RMPI-1640 (Gibco). Both culture mediums were supplemented with 10% fetal bovine serum (Cytiva), 1% Penicillin/Streptomycin, 1% Non-Essential Amino Acids, 1% Sodium Pyruvate, 1% HEPES, and 1% Glutamax. All supplements aside from FBS were obtained from Gibco. MC38 cells were tested for mycoplasma contamination prior to use and all cells were cultured at 37°C with 5% CO_2_.

### 2.3 PBMC isolation from healthy donors

Peripheral Blood Mononuclear Cells (PBMCs) from healthy donors were provided by Austin We Are Blood Association with approval from the University of Texas at Austin’s Internal Review Board’s (IRB) (IRB#2018-04-0006). Cells were flushed from a leukocyte reduction system cone according to protocols outlined previously (28). PBMCs were then isolated from the blood using Stem Cell Sep Mate Tubes (SepMate 85450). Freshly collected blood was diluted with PBS and gently loaded onto a 50 ml SEPMATE™ tube containing 15 ml of Lymphoprep density gradient (Stem Cell 04-03-9391101). Cells were collected according to the SEPMATE™ PBMC Isolation protocol and resuspended in freezing media (45% RPMI/45%FBS/10%DMSO). Cells were then frozen at 1°C per hour overnight in a -80°C freezer using a Mr. Frosty™ Freezing Container and stored in liquid nitrogen. All samples analyzed were thawed at 37°C for 5 minutes and washed for all studies described below.

### 2.4 Patient sample analysis

Blood samples were collected from patients with advanced solid tumors enrolled in a phase 1 dose-escalation clinical trial (Clinical Trial: NCT03931681). All human subjects were assessed for medical decision-making capacity using a standardized, approved assessment and voluntarily gave informed consent before being enrolled in the study and the protocol was approved with institutional review board approval (IRB# 18-1816). Patients were administered 60 mg to 450 mg of OKI-179. Blood samples used for PBMC analysis were collected just prior to treatment (baseline), 2 hours, 8 hours and 24 hours (each +/- 20 minutes) after receiving their first dose of OKI-179. Patient blood samples were drawn into BD Vacutainer^®^ CPT™ Tube that were then centrifugated and processed to obtain PBMCs according to the manufacturer’s protocol. Once isolated and washed, PBMCs were resuspended in 2 mL of freezing media (45%RPMI/45%FBS/10% DMSO) and were stored overnight in a Corning CoolCell Container unit placed at -80°C to freeze at a controlled rate of 1°C per hour and then transferred to a liquid nitrogen tank for storage. Samples of all timepoints for each individual patient was thawed, stained, and analyzed on the same day for side-by-side evaluation as described below.

### 2.5 Flow cytometry - antibodies and reagents

Flow cytometry antibodies were obtained from BioLegend and Cell Signaling Technology. For surface staining, the cells were washed with flow wash buffer (FWB) containing PBS/2% FBS/5 mM EDTA and stained with surface markers. Cells evaluated only for surface proteins were then re-suspended in FWB containing the viability dye propidium iodide (PI) (Anaspec Inc AS-83215) to exclude dead cells. All samples evaluated for intracellular proteins were stained with the fixable viability dye Zombie NIR™ (BioLegend 423106) together with surface markers prior to fixation/permeabilization followed by intracellular staining at 4°C for 30min. For samples evaluated for intracellular cytokines, granzymes and acetylated H2K27 (Ac-H3K27), cells were fixed and permeabilized with BD Cytofix/Cytoperm™ kit (RRID: AB_2869008) following the manufacturer’s protocol. For FOXP3 and Ki67 intracellular staining, cells were fixed and permeabilized using the Foxp3/Transcription Factor staining Buffer Set (Thermo Fisher Scientific 00-5523-00). All samples were then run on a Cytek Aurora spectral cytometer (5L 16UV-16V-14B-10YG-8R). Briefly, spectral channels were unmixed on the SpectroFlo^©^ software using fluorophore-specific single color controls using compensation beads (ThermoFisher 01-2222-41) and cells for viability dyes. All post-acquisition analysis was performed using FlowJo software (v10.3).

Flow cytometric analysis was performed with the following antibodies that all were obtained from BioLegend unless otherwise indicated, for staining human cells: CD3 (OKT3), CD4 (SK3), CD8 (RPA-T8), CD95 (DX2), CD45RA (HI100), CCR7 (G043H7), CD16 (3G8), CD14 (M5E2), HLA-DR (I.243), CD19 (SJ25C1), CD11b (ICRF44), CD56 (5.1H11), CD127 (A019D5), CD28 (CD28.2), CD62L (DREG-56), IL-2 (MQ1-17H12), TNFα (MAB11), IFNγ (4S.B3), IL-4 (MP4-25D2), IL17 (BL168), Ki67 (KI67), ICOS (C398.4A), FOXP3 (206D), Granzyme B (QA18A28) Ac-H3K27 (Cell Signaling Technology; Clone: 15562S), TCF1/7 (Cell Signaling Technology; Clone: 8490S), LEF1 (Cell Signaling Technology; Clone: 90511S); mouse cells: CD45 (30-F11), CD5 (53-7.3), CD4 (GK1.5), CD8 (SK1), CD44 (IM7), NK1.1 (PK136), CD11b (M1/70), H2kb (34-1-2S), IL-2 (JES6-5H4), TNFα (MP6-XT22), IFNγ (XMG1.2), Ac-H3K27 (Cell Signaling Technology; Clone: 15562S), TCF1/7 (Cell Signaling Technology; Clone: 8490S)

### 2.6 *In vitro* and *ex vivo* acetylation level analysis

For *in vitro* evaluation of histone 3 lysine 27 acetylation (H3K27-Ac) levels, PBMCs from healthy donors were thawed at 37°C for 5 minutes, washed and resuspended in complete RPMI. Cells were counted on a hemocytometer using trypan blue to exclude dead cells, then plated at a density of 200,000 cells/well in a 96-well round bottom plate. Cells were treated with either vehicle control (DMSO), OKI-005, panobinostat or a SHP2 inhibitor (SHP099) as indicated. After incubation, cells were then stained for surface proteins with a fixable viability marker for 20 minutes at room temperature followed by washing and evaluated for intracellular histone acetylation (Ac-H3K27) for 30 minutes at 4°C. Panobinostat was chosen as our positive control because it is a potent, pan-HDACi expected to induce robust acetylation and also provide as comparison to another class of HDACi for assessing function. Conversely, we chose SHP099 as a negative control as it is a SHP2 inhibitor with no known effects on HDAC activity.

For *ex vivo* analysis of acetylation in patients, PBMCs collected at each time point were immediately analyzed after thawing for surface markers and intracellular Ac-H3K27 alone or together with TCF1/7 and LEF1 for 30 minutes at 4°C where indicated. For patient analysis, samples collected at all timepoints were stained side-by-side and analyzed together to compare Ac-H3K27 MFI level change by normalizing to baseline using the following formula: ((X_timepoint_ – X_baseline_)/(X_baseline_))*100.

### 2.7 *In vitro* T cell assays with healthy donor PBMCs

PBMCs were thawed at 37°C for 5 minutes, washed and resuspended in complete RPMI. Cells were counted on a hemocytometer using trypan blue to exclude dead cells, then plated at a density of 200,000 cells/well in a 96-well round bottom plate. For intracellular cytokine analysis, cells were stimulated with 1X of BioLegend Cell Activation Cocktail (81 nM Phorbol-12-yristate 13-acetate (PMA); 1.34 μM Ionomycin). After 1 hour, brefeldin A (BD GolgiPlug Protein Transport Inhibitor; 555029) was added and cells were analyzed 18 hours later by flow cytometry using methods described above. For proliferation analysis, PBMCs were stained with Tag-it Violet™ Proliferation and Cell Tracking Dye (625 nM, BioLegend 425101) for five minutes at 37°C and washed with cold media. T cells were then negatively enriched using MojoSort™ Human CD3 T Cell Isolation Kit (BioLegend 480131). Cells were stimulated with DynaBeads (ThermoFisher 1161D) at 37°C for 72 hours in the presence or absence of inhibitors as indicated.

### 2.8 Murine *in vivo* colorectal models

Female C57BL/6J Wild-Type (RRID: ISMR_JAX:000664) mice were obtained from Jackson Laboratories. MC38 cells (1 x10^6^ cells) resuspended in 0.1-ml of HBSS with a final dilution of 30% Corning Matrigel Matrix were injected subcutaneously in the right flank. When average tumor size reached 100 mm^3^, mice were randomized to treatment groups and treated with either vehicle administered orally, OKI-179 (60 mg/kg/day) administered orally, αPD-1 (RMP1-14, BioXCell BE0146) administered intraperitoneally (i.p.) (250 μg), or a combination of OKI-179 at 60 mg/kg (oral) and αPD-1 as indicated. Animals were euthanized according to IACUC protocol (AUP-2021-00146) when tumor size reached 1500 mm^3^ or due to tumor ulceration.

For *ex vivo* acetylation analyses, mice were bled by tail-vein treated with either vehicle control or OKI-179, 2 or 24 hours prior. Samples were then treated with RBC Lysis Buffer (BioLegend 420302) according to the manufacturer’s protocol. Cells were then stained for surface proteins and a viability dye, followed by fixation and intracellular staining for Ac-H3K27 similar to protocols described above. For cytokine analysis, splenocytes were isolated from mice at the indicated time points and treated with RBC Lysis Buffer (BioLegend 420302) according to the manufacturer’s protocol. The cells were then counted, plated at a density of 200,000 cells/well and stimulated with PMA/Ionomycin in the presence of BFA overnight for intracellular cytokine (IL-2, IFNγ and TNFα) analysis.

### 2.9 MC38 cell viability assays and phenotypic analysis

MC38 cells were seeded into a 96-well, white solid bottom assay plate at 5,000 cells/well in 200 µl of medium. Cells were treated with the indicated concentration of OKI-005 or panobinostat. After 72 hours, viability was determined using CellTiter-Glo ^®^ assays (Promega G7570) and analyzed using a BioTek Synergy H1 Microplate Reader. For immune phenotype changes, MC38 cells were seeded into a 48-well, clear solid bottom assay plate at 100,000 cells/well in 1 ml of medium. Cells were treated at indicated concentrations of OKI-005 while cells treated 50 ng/ml of recombinant murine IFNγ (positive control, ThermoFisher PHC4031) for 24 hours and stained for flow cytometric analysis as described above.

### 2.10 Statistical analyses

Normalization for the indicated graphs was calculated using the following formula: X_normalized_ = ((X_V_ – X_Control_)/(X_Control_))*100. Statistical analyses were performed with Prism (v9; GraphPad Software) to determine statistical significance as indicated using either a one sample Student *t* test, paired Student *t* test, repeated measures 1-way ANOVA for normally distributed data or Kaplan-Meier curves using the log-rank test.

## 3 Results

### 3.1 Sustained HDAC inhibition during stimulation directly alters T cell cytokine production

**Figure 1:**
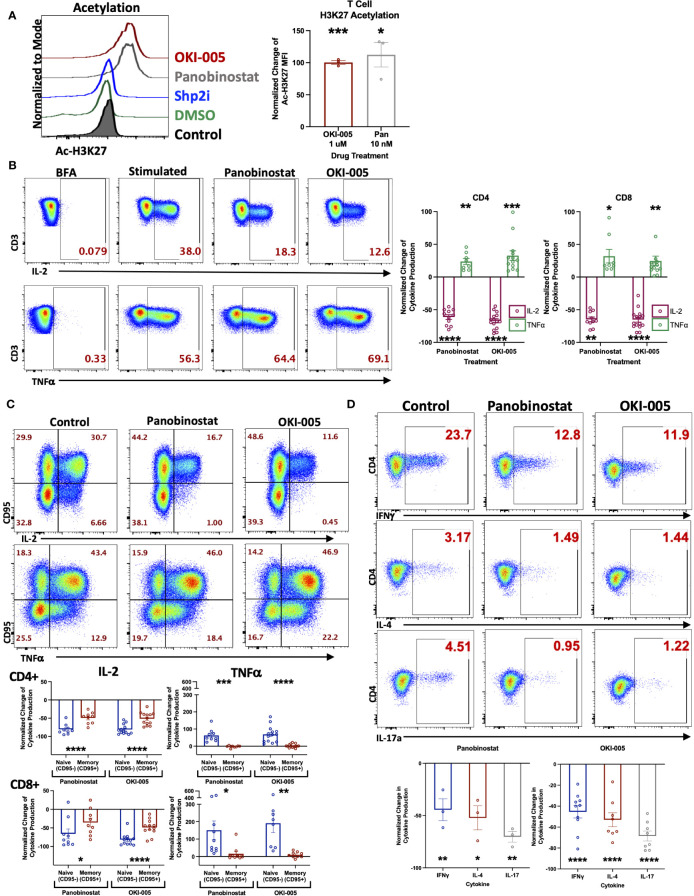
OKI-005 rapidly induces acetylation in T cells and differentially impacts cytokine production *in vitro*. **(A)** Healthy donor PBMCs were treated with DMSO, 1 µM OKI-005, 10 nM of Panobinostat, or 5 µM with a SHP2i (SHP099). After 24 hours, acetylation was analyzed by flow cytometric staining and quantification of intracellular Ac-H3K27 levels. Shown are representative flow plots (left) of the Mean Fluorescent Intensity (MFI) of Ac-H3K27 gated on CD3+ T cells from one of three independent experiments. Graph (right) shows cumulative results using the normalized MFI of Ac-H3K27 in treated T cells compared to DMSO-treated control cells. **(B)** PBMCs were stimulated with PMA/Ionomycin for 18 hours in the presence of DMSO, 10 nM of Panobinostat, or 1 μM of OKI-005. Cytokine production of IL-2 and TNFα were then evaluated by intracellular cytokine staining and flow cytometric analysis. Shown are representative flow cytometry plots of cytokine production gated on CD3+CD4+ T cells that are from one of eleven independent experiments. Cytokine production of IL-2 and TNFα were quantified in CD3+CD4+ and CD3+CD8+ T cells and graphed; n=11. **(C)** PBMCs were stimulated as described in **(B)** and quantified in memory (CD95+) and naïve (CD95-) CD3+CD4+ and CD3+CD8+ subsets separately. Shown are representative FACs plots from one of ten independent experiments; n=10. **(D)** PBMCs were stimulated as described in **(B)**. Cytokine production of IFNγ, IL-4, and IL-17 were then evaluated by flow analysis. Shown are representative flow cytometry plots gated on CD3+CD4+ T cells from one of at least three independent experiments; n>3. Graphs show cumulative results with bar representing the mean ± SEM and each symbol representing results from independent experiments of the frequency of the indicated cytokine production in T cells normalized to experimental controls. Statistical analysis was performed using an unpaired Student’s t-test **(A, B, D)** and a paired Student’s t-test **(C)**; *P<0.05, **P<0.01, ***P<0.001, **** P<0.0001.

Prior investigations with OKI-179, a potent class-I HDACi, have demonstrated the ability to enhance anti-tumor immune responses to treatment with αPD-1 (16, 27, 29). However, the direct effects on T cells were not well characterized. To accomplish this, we used OKI-179 and OKI-005, which are largazole derivatives that serve as pro-drugs to the active compound OKI-006, which previous studies have reported to inhibit class I HDACs 1,2,3 (IC_50 = _1.2, 2.4, 2.0 nM, respectively), and no activity towards class IIa HDACs (16). OKI-179 has been shown to have more favorable properties for use *in vivo* while OKI-005 is better suited for *in vitro* evaluation, where it is rapidly converted to its active metabolite. Thus, OKI-179 was used for all *in vivo* studies whereas OKI-005 was used for *in vitro* assays (16, 27). Therefore, to verify that T cells are directly responsive to HDAC inhibition, we initially performed *in vitro* analyses using OKI-005 to optimize a flow cytometric assay for assessing changes in levels of acetylated histone H3 on lysine 27 (Ac-H3K27) to use as a readout for T cell responsiveness to HDACi based on previously reported protocols (30). As shown in **Figure 1A** **(**gating strategy shown in **Supplementary Figure S1**), OKI-005 induced robust increases in Ac-H3K27 levels in T cells similar to that of cells treated with the non-specific inhibitor panobinostat, which served as a positive control. The specificity of this on-target effect was verified by the lack of acetylation changes in cells treated with an inhibitor of a non-HDAC protein, SHP099 (SHP2i) that served as a negative control. Next, we evaluated the impact of OKI-005 on T cell function by assessing changes in cytokine production and found that T cells’ ability to produce IL-2 was impaired (**Figure 1B**). Interestingly, not all cytokines were suppressed similarly, as the frequency of TNFα producing-cells increased in response to OKI-005 as well as panobinostat. Given that TNFα was increased, these results indicate that the decrease in IL-2 production was not attributed to T cell apoptosis and that HDACi induces cytokine-specific responses. Furthermore, careful evaluation of T cells suggested that these effects were not global, as not all cells lost the ability to produce IL-2 in both CD4 and CD8 T cells.

The differential responses of individual cells’ cytokine production to OKI-005 led us to consider subset-specific effects given the important role epigenetics play in T cell differentiation and imprinting effector functions during TCR activation by antigens (31). Therefore, we next assessed cytokine production in relation to expression of CD95, which is expressed by all memory subsets, to determine whether changes were restricted preferentially in naïve T cells (CD95-) or memory T cells (CD95+) (32). This analysis revealed that IL-2 loss was most prominent in naïve T cells, whereas memory cells displayed modest decreases in comparison (**Figure 1C**). This subset specific response to HDACi was even more pronounced when evaluating TNFα, which was almost completely restricted to naïve T cells; with no discernable gain by memory cells (**Figure 1C**).

Considering the subset-specific responses between naïve and memory cells, as well as cytokine-specific changes of IL-2 and TNFα, we next sought to determine whether HDACi uniquely affects cytokine production by specific CD4 T helper (Th) subsets, given how epigenetics imprint lineage-specific gene expression patterns which confer distinct effector functions. To do this, we determined whether OKI-005 differentially affects the production of cytokines that define specific CD4 Th subsets: IFNγ (Th1), IL-4 (Th2) and IL-17A (Th17). Unlike differences between naïve and memory subsets, OKI-005 and panobinostat both suppressed cytokine production similarly across all CD4 Th subsets (**Figure 1D**). Altogether, these results demonstrate that while both OKI-005 and panobinostat were mostly suppressive, these effects differed between cytokine analyzed and the subsets evaluated.

### 3.2 Disconnect between T cell responses in patients and *in vitro*

**Figure 2:**
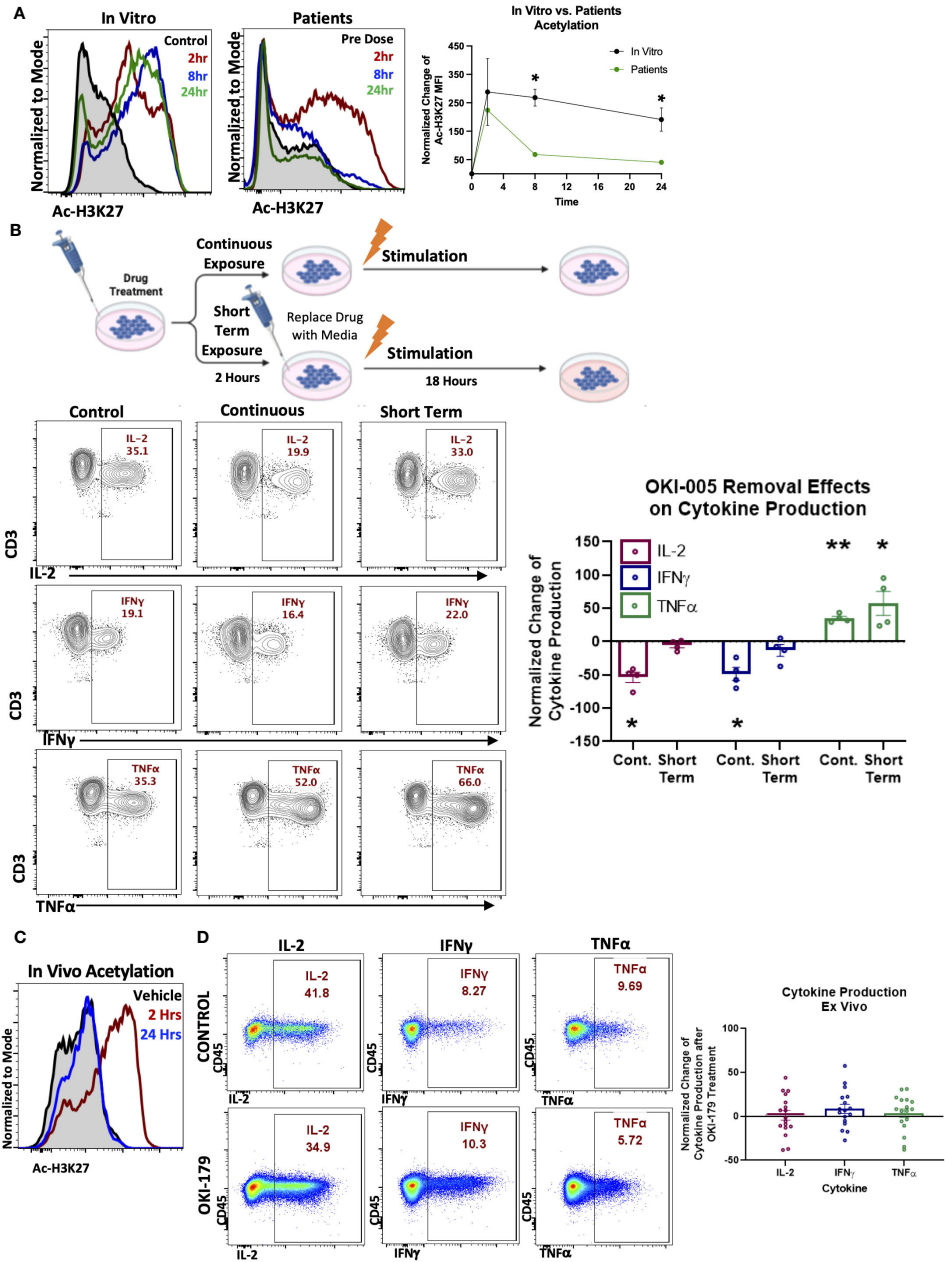
T cell acetylation upregulation in patients is transient and effects are dynamic and rapidly reversible. **(A)**. (Left) PBMCs were treated with 1 μM OKI-005 *in vitro* for indicated time periods and assessed for Ac-H3K27 using flow cytometry intracellular staining. (Right) PBMCs were taken from patients receiving OKI-179 in the clinic, before the first dose (baseline), 2 hours post-dose, 8 hours post-dose, and 24 hours post-dose and assessed for Ac-H3K27 using flow cytometry. Representative patient shown was dosed with 200 mg of OKI-179. Flow cytometric histograms are representative of 32 individual patients receiving OKI-179. MFI of Ac-H3K27 was quantified and normalized to DMSO-treated control cells for *in vitro* analyses or baseline samples from corresponding patients for *ex vivo* analyses. Graph shows cumulative data of *in vitro* results normalized to controls within each experiment; n=4. **(B)** Schematic created by Biorender.com displaying drug treatment exposure. PBMCs were treated with OKI-005 at 1 μM for 2 hours. OKI-005 was either removed prior to stimulation (Short term exposure) or retained in the media (Continuous exposure) and cells were stimulated with PMA/Ionomycin and assessed for cytokine production after 18 hours. Production of IL-2, TNFα, and IFNγ, was assessed and percent of cytokine producing total T cells was quantified and normalized to DMSO-treated stimulated controls; n=4. **(C)** C57Bl/6 mice treated with vehicle (control) or OKI-179 (60 mg/kg). Representative histogram of T cells extracted from blood and analyzed for Ac-H3K27 immediately *ex vivo*. **(D)** Representative flow plots of splenocytes extracted 2 hours following treatment that were stimulated *ex vivo* overnight and evaluated for IFNγ, IL-2, and TNFα production. Frequency of T cells producing each cytokine were quantified and normalized to control mice for each experiment; n=4 mice per group for 4 independent experiments. Graphs show cumulative data of results normalized to controls within each experiment for frequencies of indicated cytokines in T cell subsets with the bar representing mean ± SEM and each symbol representing results from independent experiments; *P<0.05, **P<0.01.

We next evaluated T cell responses in patients enrolled in a dose-escalation phase-I clinical trial by assessing changes in Ac-H3K27 (28). Previous pharmacokinetic results of other HDACi in patients have shown that compound levels typically peak in the serum within hours and decrease over 24 hours following treatment (21, 33). To capture T cell responses during this critical timeframe, blood samples were collected before (baseline) and 2-, 8-, and 24-hours following their first dose of OKI-179. Evaluation of Ac-H3K27 levels in T cells at these timepoints revealed that T cells rapidly respond to OKI-179 as acetylation levels peaked within 2 hours and began to wane within 24 hours (**Figure 2A**). Importantly, the pharmacodynamics of acetylation observed in T cells mirror that of the pharmacokinetics of OKI-179 as indicated by changes in serum levels of the active compound, OKI-006, that peak at 2 hours and decrease over the subsequent 24 hours, as previously reported (28, 34). Although results from only one representative patient is shown here, similar kinetics in acetylation and compound levels following their first dose were observed across all patients (n=32) (28, 34). Because the dynamic changes in OKI-006 levels observed in patients likely do not occur *in vitro*, we next compared changes in acetylation levels in similar time courses. In contrast to the transient responses observed in patients, acetylation levels in T cells remained high over 24 hours *in vitro* (**Figure 2A**).

These critical differences in drug exposure likely have important implications related to T cell function that could explain the discrepancy between suppression found *in vitro* and the ability of HDACi to enhance anti-tumor T cell responses *in vivo* using murine models (19, 35, 36). To determine this, we developed an *in vitro* model by removing OKI-005 after 2 hours followed by stimulation to better mimic OKI-179 effects on T cells in patients, as depicted in **Figure 2B**. In contrast to T cells continuously exposed to high concentrations of OKI-005, where IL-2 production was significantly decreased as previously observed (**Figure 1B**), the ability of T cells to produce IL-2 was unaffected in T cells exposed to short-term HDACi (**Figure 2B**). Similarly, evaluation of proliferation found that T cell proliferation was abrogated only when stimulated in the continuous presence of OKI-005 (**Supplementary Figure S2**). Surprisingly, the increase in TNFα production observed during continuous drug exposure with stimulation occurred in conditions where the compound was removed and even trended towards further enhancement (**Figure 2B**). Collectively, these results indicate that while HDACi can inhibit cytokine production by T cells *in vitro*, critical differences in the kinetics in drug exposure in patients need to be taken into account.

### 3.3 Evaluation of cytokine production *in vivo* to verify T cell responses

Given the reversibility of OKI-005’s effects *in vitro*, we next examined these findings *in vivo* by evaluating T cell cytokine production from mice treated with OKI-179 or vehicle control. First, we verified that the murine model recapitulates the pharmacokinetics of the drug observed in patients (27) by evaluating T cell Ac-H3K27 levels in blood samples collected 2 and 24 hours following treatment with OKI-179 compared to vehicle control treated mice. As shown in **Figure 2C**, T cell responses to OKI-179 in mice exhibited high Ac-H3K27 levels observed after 2 hours that mostly recedes within 24 hours, as previously reported (27). Importantly, T cells collected 2 hours post treatment displayed no significant changes in cytokine production when stimulated *ex vivo* (**Figure 2D**; **Supplementary Figure S3**). Thus, T cell suppression observed using *in vitro* assays shown here (**Figure 1**) and in previous reports (25, 37, 38) is due to continuous exposure to inhibitors during stimulation that does not reflect the physiological effects observed in patients. Altogether these findings indicate that while HDACi can suppress T cells, these effects are likely only transient in patients, due to the dynamics in compound levels in serum analysis that correlate with acetylation changes that spike and recede.

### 3.4 HDAC treatment in patients results in rapid but transient acetylation in TCF1/7+ subsets

**Figure 3:**
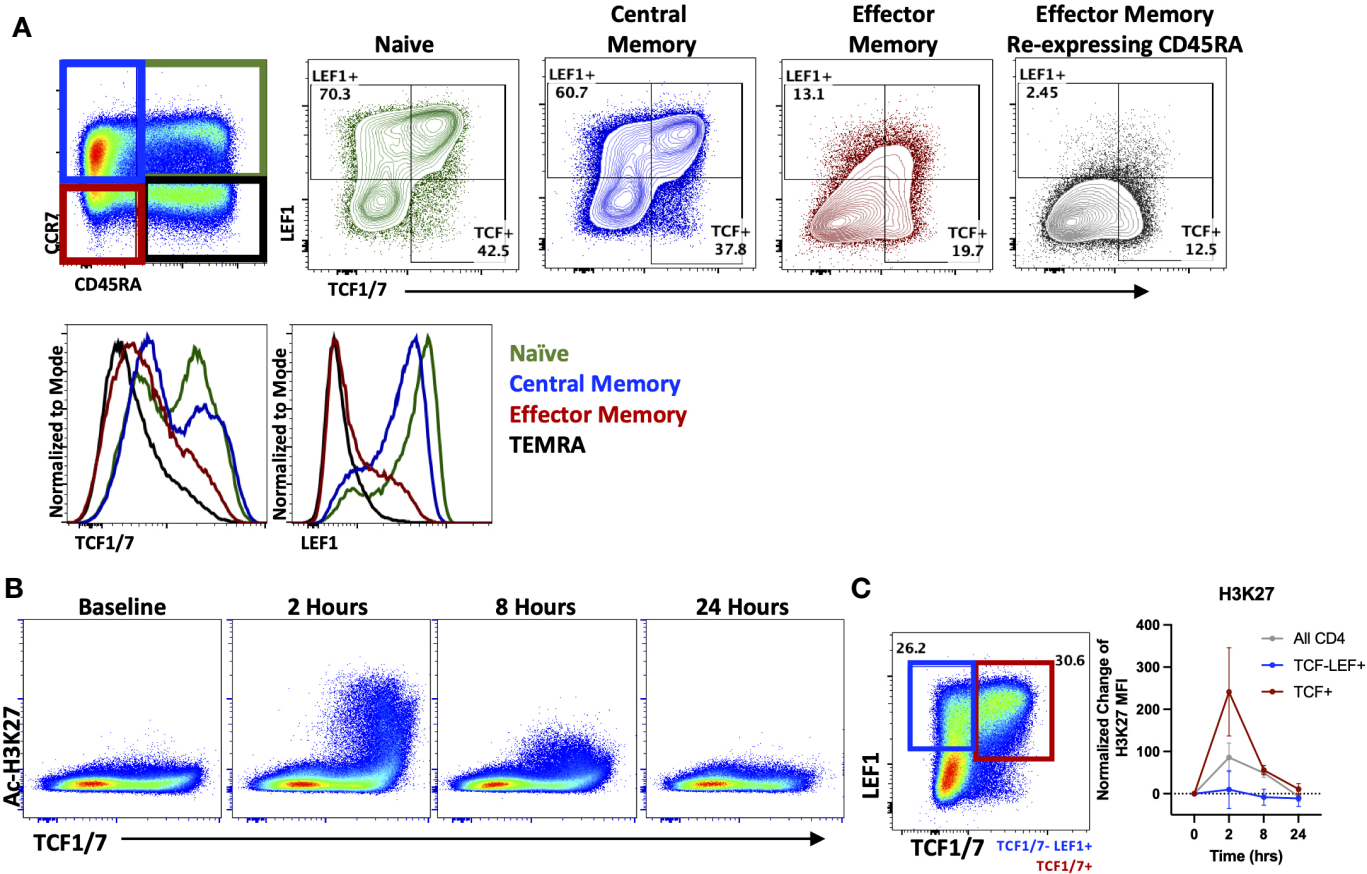
OKI-179 alters acetylation in TCF1/7+ subsets. **(A)** T cells were gated based on CD45RA and CCR7 expression and assessed for TCF1/7 and LEF1 expression as shown. Histograms are representative of 6 individual patients receiving OKI-179. **(B)** Flow cytometry plots display changes in Ac-H3K27 in patient PBMCs taken at indicated time points after the initial dose of OKI-179 in comparison to TCF1/7 expression. **(C)** T cells were gated based on TCF1/7 and/or LEF1 expression as shown. MFI of Ac-H3K27 was quantified in TCF1/7+, LEF+TCF1/7-, and total CD4+ populations. Graphs show cumulative data of timepoints normalized to baseline controls within each patient; n=6 patients.

As seen in **Figure 1C**, HDACi differentially affected naïve and memory T cell populations when evaluating cytokine production *in vitro*. Therefore, we next analyzed the effect of OKI-179 in patients to determine whether HDACi impacts distinct T cell subsets *in vivo* as well. To do this, we evaluated changes in Ac-H3K27 levels in relation to expression of the transcription factors, T cell factor 1 (TCF1/7) and lymphoid enhancer-binding factor 1 (LEF1) as they possess HDAC domains themselves and engender “stemness” in naïve and some memory subsets that is lost over prolonged periods of activation (39–41). In alignment with prior characterization of TCF1/7 and LEF1 expression in T cell subsets (42), our analysis of patients’ T cells showed high expression of TCF1/7 and LEF1 in naïve (CD45RA+ CCR7+) and central memory (CD45RA- CCR7+) populations compared to effector memory (CD45RA- CCR7-) and effector memory re-expressing CD45RA (CD45RA+ CCR7-) subsets (**Figure 3A**). We first looked at Ac-H3K27 upregulation in relation to TCF1/7 and found that acetylation was predominantly observed in TCF1/7+ cells (**Figures 3B, C**). However, no correlation was found with LEF1 when breaking down subsets further based on TCF1/7 and LEF1 expression (**Figure 3C**). Altogether these results indicate that T cells are not uniformly responsive to OKI-179.

### 3.5 Frequencies of T cells among circulating PBMCs is transiently increased following HDACi treatment

**Figure 4:**
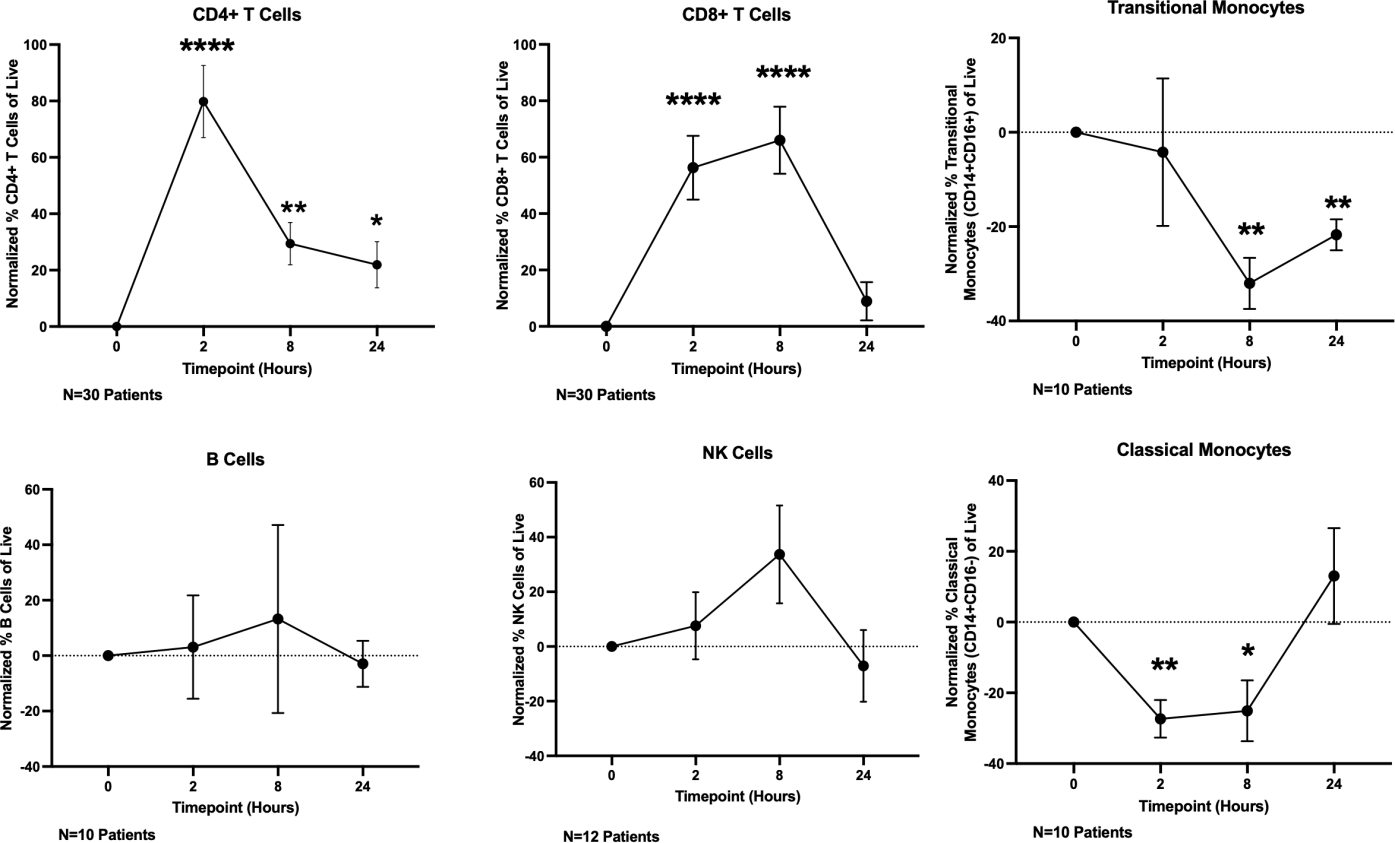
OKI-179 induces rapid changes in immune subset frequencies in the blood of patients. PBMCs were taken from patients receiving OKI-179 in the clinic, at baseline, 2 hours post-dose, 8 hours post-dose, and 24 hours post-dose and assessed for immune subset frequencies. Statistical analysis was performed using an unpaired student t-test with GraphPad PRISM 9 software. Results are expressed as mean ± SEM; *P<0.05, **P<0.01, ****P<0.0001.

Having demonstrated that T cells directly respond to OKI-179 shortly following treatment as indicated by acetylation changes, we next determined whether OKI-179 alters the frequency of T cells and other immune subsets in the blood by flow cytometric analysis. Using the gating strategy shown in **Supplementary Figure 4**, these time course analyses revealed T cell frequencies significantly increased among PBMCs shortly following treatment (**Figure 4**; **Supplementary Figure S4**). This increase does not appear to be attributed to a short burst in proliferation as circulating CD4 and CD8 T cells showed minimal changes in the expression of proliferation marker Ki67 (**Supplementary Figure S5**). Whether the increase in T cell frequency among PBMCs is due to an increase in absolute numbers in the blood or as a result of a concomitant decrease in monocytes is unknown as absolute cell counts were not performed in this analysis. Nevertheless, these results demonstrate that immune subset composition is significantly and transiently altered within hours following treatment with OKI-179 in patients.

### 3.6 Effects of OKI-179 on specific T cell subsets in patients

**Figure 5:**
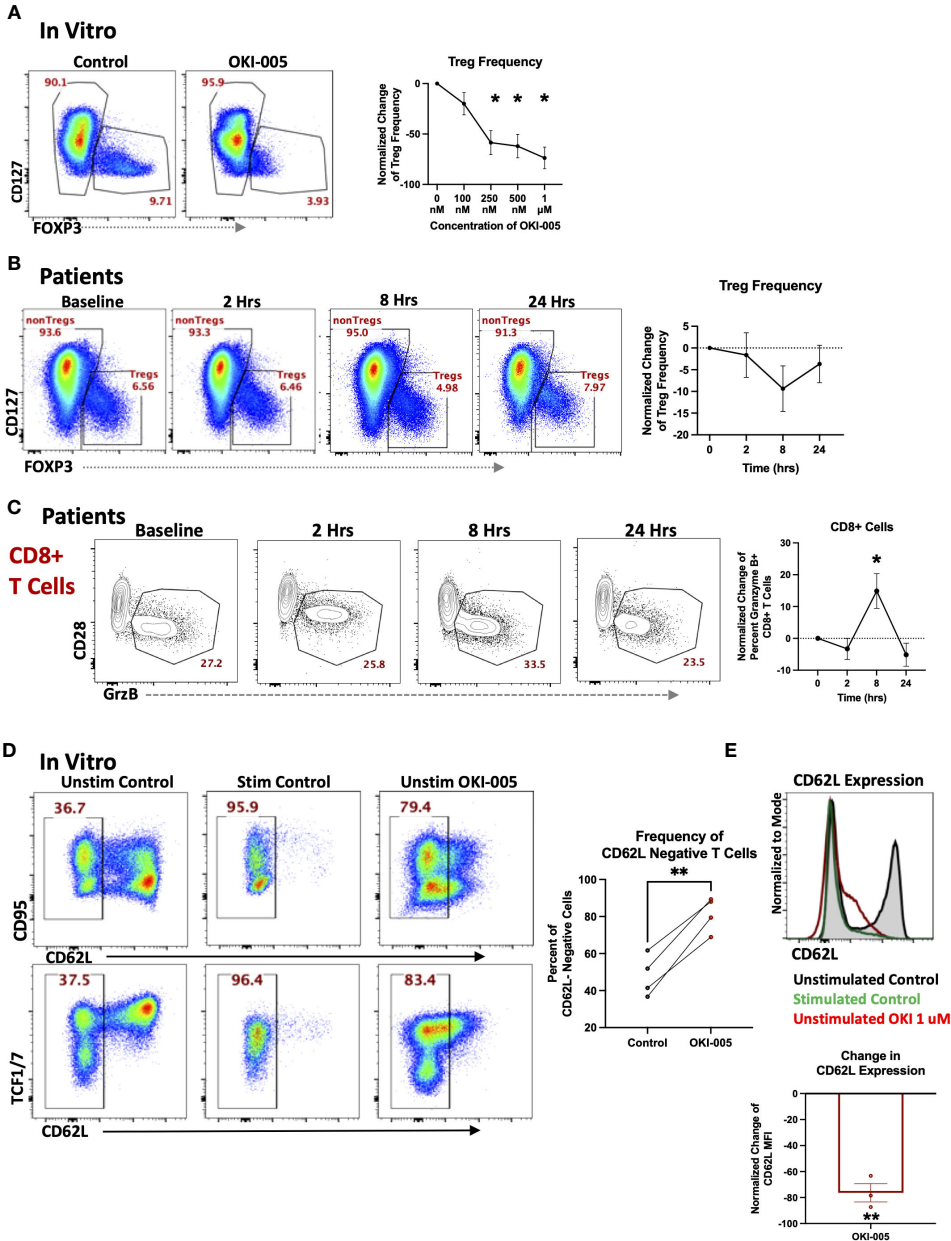
OKI-005/OKI-179 affects Treg and cytotoxic T cell subsets. **(A)** PBMCs were treated with OKI-005 at doses from 0 to 1 μM for 24 hours and assessed for CD4, CD25, CD127, and FOXP3 expression. Flow cytometry plots and histograms are representative of three independent experiments. Percent of FOXP3+ CD4 T cells were quantified and normalized to DMSO-treated control cells and MFI of FOXP3 was quantified and normalized to DMSO-treated control cells; n=3; **(B)** PBMCs isolated from patients receiving OKI-179 were assessed *ex vivo* for FOXP3 expression. Representative flow cytometry plots are gated on CD3+CD4+ T cells. Percent of FOXP3+ CD4 T cells were quantified and normalized to baseline samples of corresponding patients; n=18. **(C)** Patient PBMCs were assessed *ex vivo* for Granzyme B within CD8+ T cells using flow cytometry. Flow cytometric plots are representative of individual patients receiving OKI-179. Percent of Granzyme B+ cells were quantified and normalized to baseline samples within the same patient and graphed; n=24. **(D)** PBMCs were treated with 1 μM of OKI-005 *in vitro* or PMA/Ionomycin (positive control) and T cells were surface stained for CD95 and CD62L expression and intracellularly stained for TCF1/7. Panels show frequencies of CD62L- cells and CD62L MFI of T cells following 18-hrs of treatment. Flow cytometry plots are representative in showing CD62L loss in relation to (top) CD95 and (bottom) TCF1/7; n=4. **(E)** CD62L expression in overlaid histograms gated on CD3+CD4+ T cells. MFI of CD62L was quantified and normalized to DMSO control; n=4. Results shown are cumulative with in the graphs expressed as the mean ± SEM; *P<0.05, **P<0.01.

In addition to changes in total T cell frequencies, we also evaluated changes in specific subsets as HDACi have been shown to affects subsets important to anti-tumor immune responses such as Tregs (23, 43, 44) and cytotoxic CD8+ T cells (9). Previous studies have shown acetylation plays a critical role in Tregs such as regulating both the transcription and stability of FOXP3 in Tregs as well as their function (44, 45). However, the impact on Treg frequencies by different HDAC inhibitors has varied, as some have shown an increase in Treg frequencies and function (46, 47), while treatment with other inhibitors have reported a decrease in Tregs (44). These differential responses by Tregs are likely attributed to the different selectivity profiles of inhibitors toward individual HDACs, which have been shown to play various roles in Treg biology (45). Therefore, we determined whether OKI-005/OKI-179 impacts Tregs by evaluating FOXP3 expression in healthy donor PBMCs after treatment with OKI-005 *in vitro*. As shown in **Figure 5A**, OKI-005 resulted in ~50% reduction in FOXP3 expression among CD4+ T cells treated with concentrations over 250 nM. Furthermore, changes in Treg frequencies appear to be driven by decreased FOXP3 expression rather than preferential apoptosis by Tregs based on MFI quantification of FOXP3 specifically among Tregs (**Supplemental Figure S6**). Although not statistically significant, longitudinal analysis of PBMCs from patients treated with OKI-179 also revealed a similar decreasing trend in FOXP3+ Treg frequencies among CD4 T cells 8 hours following treatment (**Figure 5B**) that, together with our results from *in vitro* analyses, indicate that Treg expression of FOXP3 is impacted by OKI-005/OKI-179.

Given the importance of CD8 T cells in mediating anti-tumor immune responses to monotherapy with other HDACi (9, 10) and previous reports of specific HDACs and HDACi in regulating the cytotoxic gene programs of CD8 T cells during development (48), we determined whether OKI-179 changed expression of the cytotoxic molecule granzyme B (GrzB) by CD8 T cells in patients. This analysis revealed an increase in the frequency of GrzB+ expressing-CD8 T cells circulating in the blood of patients within 8 hours following treatment (**Figure 5C**). To gain insight into whether this change was due to directly upregulating GrzB on a per cell basis, we evaluated the direct effects of OKI-005 on granzyme expression in T cells following treatment *in vitro* but found no changes in GrzB expression among CD8 T cells (**Supplementary Figure S7**).

### 3.7 CD62L expression is directly regulated by HDACi treatment

Considering that the increase in GrzB+CD8 T cell frequencies occurring within patients following treatment did not appear to be attributed to HDACi-induced GrzB upregulation on a per cell basis, based on *in vitro* studies, we postulated that some changes in the blood could be attributed to altered trafficking of T cells already expressing GrzB. This is supported by our previous findings of increased T cell frequencies that occurred within hours, a result likely not due to a burst in proliferation as indicated by no change in Ki67+ cells. Furthermore, prior analyses with other HDACi have shown changes in expression of key homing molecules following treatment (25). This includes CD62L, which plays an important role in regulating T cell retention in lymph nodes as well as trafficking to other tissues (49). Therefore, we determined whether treatment directly altered CD62L levels on T cells by treating PBMCs with OKI-005 *in vitro.* As shown in **Figures 5D, E** we observed a drastic decrease in CD62L levels on the cell surface of both naïve and memory T cells akin to CD62L shedding during T cell receptor activation (50). Together these results suggest that some subset-specific changes observed in patients’ blood following treatment with OKI-179 could be due to altered T cell trafficking.

### 3.8 OKI-179 enhances anti-tumor immune responses to immunotherapy with αPD1

While we have shown OKI-005/OKI-179 directly affects immune cells in patients, HDACi’s effects on tumor immunogenicity is also key in its efficacy (8). Based on these previous findings of OKI-179 and the direct immune effects shown here, we sought to identify a tumor model in which OKI-179 was effective and immune-dependent. Therefore we chose to use the solid tumor model of colorectal cancer MC38, as previous investigations have shown immune-dependent responses to both a class-I selective and pan-inhibitor (6, 8). We analyzed OKI-005 effects *in vitro*, which found OKI-005 had little direct impact on MC38 tumor growth except at higher concentrations (**Supplementary Figure S8A**), but showed increased expression of MHC-I on tumor cells (**Supplementary Figure S8B**), which indicates changes in immunogenic antigen presentation that may also play a role in anti-tumor responses, and is consistent with prior work in B cell lymphoma (16). In our own investigation, B cells displayed increases in expression of MHC-II when PBMCs were treated *in vitro* (**Supplementary Figure S9A**). This was also true in patients as MHC-II was upregulated on both B cells and classical monocytes 8 hours following treatment (**Supplementary Figures S9B, C**). Altogether, these findings indicate that OKI-005/OKI-179 regulates the immunogenicity of MC38 tumor cells.

**Figure 6:**
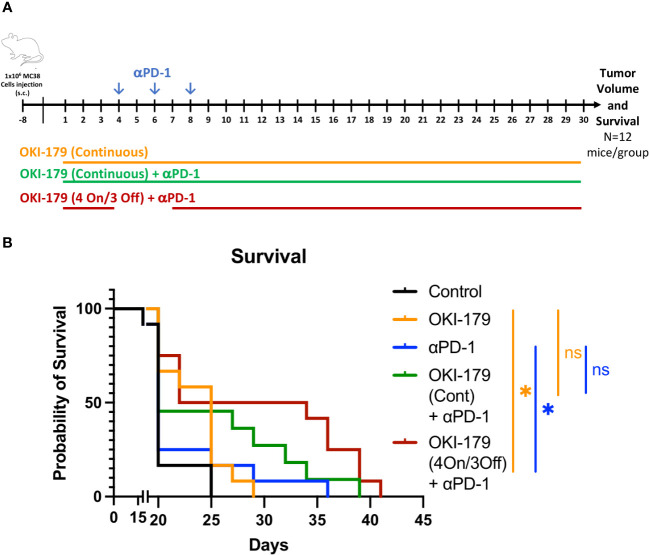
Effect of OKI-179 in murine colorectal cancer model. **(A)** Schematic of treatment schedule of C56BL/6 mice (n=12 mice/group) inoculated with 1x10^6^ MC38 tumor cells. Treatment began when tumor was established (~9 days after inoculation). Mice were treated with either vehicle (citrate buffer), OKI-179 (60 mg/kg) alone for 30 days, αPD-1 (250 μg) three times a week (as indicated), or a combination as indicated with continuous OKI-179 (60 mg/kg) or an on/off OKI-179 dosing schedule. **(B)** Overall survival of mice treated with vehicle, OKI-005 (orally), αPD-1, or a combination for 30 days. *P < 0.05; ns, Not statistically significant; compared with either OKI-179 alone (p=0.0162) or αPD-1 alone (p=0.0137).

The enhancement of MHC expression by both tumor and antigen presenting cells following treatment is likely partly responsible for enhancing responses to immunotherapy with αPD-1 (16, 29). Collectively, these results suggest that OKI-179 primes the TME by enhancing tumor immunogenicity and altering T cell trafficking. However, our analysis also indicates that OKI-005 directly inhibits T cell cytokine production if present continuously during stimulation. Therefore, we ascertained whether stopping OKI-179 dosing during immunotherapy administration would prove beneficial in anti-tumor responses by priming the TME while allowing immunotherapy to fully activate T cells. Given that a four-days-on with three-days-off dosing schedule was used for some of the patients in the phase-I clinical trial due to toxicity (28), we tested whether providing this three day break during immunotherapy would enhance anti-tumor responses when compared to combination therapy without a break (**Figure 6A**). Indeed, we found that the on/off combination therapy had statistically significant enhanced survival when compared to mice treated with either OKI-179 alone or αPD-1 alone (**Figure 6B**; **Supplementary Figure S10**). In contrast, while continuous combination regimen did appear to prolong survival in some mice, it was not statistically significant when compared to either single treatment. Altogether, these findings indicate that OKI-179 enhances tumor immunogenicity and that scheduling could be important when combining an HDACi with αPD1.

## 4 Discussion

HDACi are currently being explored in the clinic to treat cancer patients and have historically been thought to exert anti-tumor effects directly on cancer cells. However, more recent studies have demonstrated an immune component in mediating response to HDACi monotherapy with multiple pan- and class I-selective inhibitors (6, 7); specifically by T cells (5, 11). Given the various roles that HDACs and epigenetic programming play in T cell biology (31), it is important to understand how distinct inhibitors directly impact T cells. Although there have been some examinations of how HDAC inhibitors directly affect T cells, the results in the literature are incomplete and often contradictory (22, 25, 38). While part of these discrepancies may be attributed to the different specificity profiles of various inhibitors towards individual HDACs, they may also be partly due to different models used, as many were performed in non-physiological conditions *in vitro*. Conversely, some investigations evaluated the effect *in vivo* that revealed increases in tumor infiltrating lymphocytes (TILs) which exhibited altered phenotypes (1, 4, 5, 10, 15) after HDACi treatment. Although informative, such approaches only provide a snapshot of T cells, as it remains unclear whether changes are attributed to direct T cell effects or secondary effects from upstream alterations, such as altered tumor immunogenicity or effects on antigen presenting cells (17, 51). Therefore, our studies sought to provide a more comprehensive picture of how these inhibitors impact T cells by evaluating responses in patients enrolled in a phase-I clinical trial receiving OKI-179 (28, 34), which has previously been shown to augment responses to immunotherapy (29).

Using a novel HDACi (OKI-179 and its cell active predecessor OKI-005) that potently, and selectively, inhibits class I HDACs, we found that HDAC inhibition has rapid dynamic effects on circulating frequencies of immune subsets. Other investigations with HDACi, evaluated changes in circulating immune subsets following treatment over the course of days (22). However, analyses of patients receiving OKI-179 demonstrated that T cell responses occur within hours, but are transient, as they wane prior to their next dose, consistent with histone acetylation and pharmacokinetic and pharmacodynamic changes in patients. These changes are likely reflective of compound exposure during this time period rather than a compensatory mechanism that alters HAT/HDAC expression or activity. This is supported by pharmacodynamic data of serum levels of the active metabolite OKI-006 (28), as acetylation changes of H3K27 in T cells mirrors the kinetics of OKI-006 availability, due to conversion from OKI-179 followed by clearance. However, this rapid response to HDAC inhibition in patients differed from T cell functional effects *in vitro* with continuous exposure. Drug washout analyses to better recapitulate patient responses showed that cytokine effects were reversible and that previous suppressive findings with high continuous drug exposure were likely due to artificial, non-physiological conditions. These findings have important implications regarding our understanding of the effects of inhibitors on T cells, as HDACi can be suppressive when the compound remains, but T cells are likely only transiently suppressed when evaluated in acute kinetic models.

Although we found that suppressive T cell effects were reversible with a washout model, continuous exposure nevertheless displayed differences between subsets *in vitro.* Consistent with differences in expression of individual HDACs in T cell subsets and epigenetics in differentiation during activation by antigen (31), our findings indicate that HDACi impact discreet subsets as IL-2 changes were predominantly contained to the naïve compartment of T cells. Furthermore, patient subsets also displayed similar differential subset effects in naïve and central memory cells compared to further differentiated T cells, seen with TCF1/7 subset breakdown. Interestingly, TCF1/7 has its own HDAC domain (40, 52). While not directly investigated in these studies, because acetylation changes may be partly attributed to inhibition of TCF1/7 HDAC activity in some populations, we observed that acetylation is predominantly altered in TCF1/7+ cells and thus needs to be explored further.

Additionally, changes to circulating T cell subsets observed in patients revealed that OKI-179 increases circulating cytotoxic cell frequencies while concomitantly decreasing Tregs, that previous evaluations of Tregs with other inhibitors conflicted on (22, 43, 44). While increases in cytotoxic cell frequencies may be attributed to alterations in T cell trafficking evaluated with CD62L expression changes, we demonstrated that decreases in regulatory T cells are not due to Treg egress. Our closed system *in vitro* model demonstrated that OKI-005 was directly altering FOXP3 expression, resulting in decreased regulatory T cell frequencies. Recent studies have shown direct effects of HDACi on FOXP3+ Tregs and implicated class IIa HDACs as key targets (47, 53) for FOXP3 repression (45). Therefore, the HDAC isoform selectivity of HDAC inhibitors and its effects on acetylation in Tregs and cytotoxic T cells needs to be further investigated to fully encompass HDACi’s mediation of anti-tumor effects. Previous studies have shown HDACs regulate acetylation of non-histone proteins. This includes acetylation of FOXP3, which has been shown to regulate FOXP3 at the protein level by altering its stability and function within Tregs (54–56). Thus, it should be noted that although changes in histone acetylation (H3K27-Ac) were used to monitor T cell responsiveness to HDACi, it is possible that modification to non-histone proteins are likely behind changes to Tregs and could also play a role in the mechanisms in altered T cell function and frequency we observed.

In addition to T cells, we also evaluated responses to OKI-179 by other immune subsets in patients. This includes increased MHC-II on both B and myeloid cells, which was further confirmed *in vitro*. Similarly, we found MHC-I upregulation on MC38 tumor cells. This suggests that in addition to direct effects on T cells, OKI-179 may also affect T cells indirectly by acting upstream on APCs. It should be noted that previous studies have shown romidepsin increased histone acetylation in PBMCs following treatment (57). Although not directly compared, romidepsin has also been shown to affect immune modulating proteins such as increasing MHC expression and can negatively impact T cell production of IFNγ (4, 58, 59). Unlike OKI-179, previous studies have reported that romidepsin results in an increase or no change FOXP3+ Tregs (22, 59). Although the timepoints are different, these results highlight potential differences between the effects of these compounds. Furthermore, romidepsin has been shown to cause in patients lymphopenia when administered as a monotherapy, while lymphopenia has not been observed in patients following treatment with OKI-179 (34, 60).

Lastly, we also evaluated the ability of OKI-179 to enhance αPD1 in the MC38 tumor model. These studies revealed that stopping treatment with OKI-179 when αPD1 treatment is started, led to enhanced responses when compared to either single treatment arm. In contrast, despite a trend toward prolonging survival, mice treated continuously with OKI-179 during αPD1 treatment was not significantly different compared to mice in either single treatment arms. Although direct comparisons of the combination groups were not statistically different, the lack of significance in the continuous combination group found in the intermittent group indicate that timing of HDACi treatment with immunotherapy should be considered. Furthermore, because some patients enrolled in the clinical trial were treated with OKI-179 on a similar on/off regimens, these findings likely have clinical implications for using this schedule when combining treatment with αPD1. While the mechanism behind the advantageous treatment break isn’t clear, these studies nevertheless highlight that scheduling could be important for combining HDACi with immunotherapy.

In summary these findings indicate that OKI-179 may prime the tumor response by enhancing tumor cell immunogenicity, increase MHC on multiple antigen presenting cell subsets, increase T cell frequencies in the blood while also altering Treg to cytotoxic T cell ratios.

## Data availability statement

The original contributions presented in the study are included in the article/**Supplementary Material**. Further inquiries can be directed to the corresponding author.

## Ethics statement

The studies involving humans were approved by Institutional Review Board at Colorado. The studies were conducted in accordance with the local legislation and institutional requirements. The participants provided their written informed consent to participate in this study. The animal study was approved by The University of Texas at Austin’s Institutional Animal Care Use and Committee (protocol AUP-2021-00146). The study was conducted in accordance with the local legislation and institutional requirements.

## Author contributions

TT: Conceptualization, Methodology, Supervision, Writing – original draft, Writing – review & editing, Project administration, Formal analysis, Data curation. NH: Methodology, Data curation, Formal analysis, Writing – original draft. AS: Investigation, Writing – review & editing. MD: Investigation, Methodology, Writing – review & editing. MS: Investigation, Methodology, Writing – review & editing. AC: Funding acquisition, Methodology, Writing – review & editing. SK: Conceptualization, Investigation, Methodology, Writing – review & editing. JR: Investigation, Methodology, Writing – review & editing. UG: Investigation, Methodology, Writing – review & editing. JD: Conceptualization, Investigation, Methodology, Project administration, Resources, Writing – review & editing. AS: Investigation, Resources, Writing – review & editing. AP: Conceptualization, Project administration, Resources, Writing – review & editing. CV: Funding acquisition, Methodology, Supervision, Writing – review & editing. SE: Funding acquisition, Project administration, Resources, Writing – review & editing.
