# Supplementary material for: Elucidating the direct effects of the novel HDAC inhibitor bocodepsin (OKI-179) on T cells to rationally design regimens for combining with immunotherapy
Source: Front Immunol. 2023 Sep 6;14:1260545. doi: 10.3389/fimmu.2023.1260545 (PMC10513502; doi:10.3389/fimmu.2023.1260545)
Supplement: Supplementary file 1 [file DataSheet_1.pdf]

Supplemental Figure 1

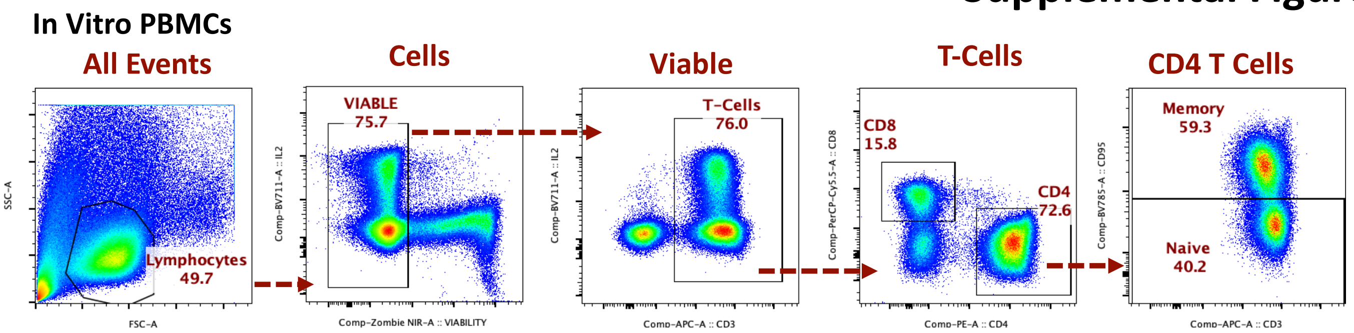

Supplemental Figure 1. Gating strategies for analyzing the immune compartment *in vitro*. Representative sequential gating schemes for evaluation of *in vitro* PBMCs from healthy donors. The initial plot in each gating scheme is gated on All events and sequential gating is indicated by arrows.

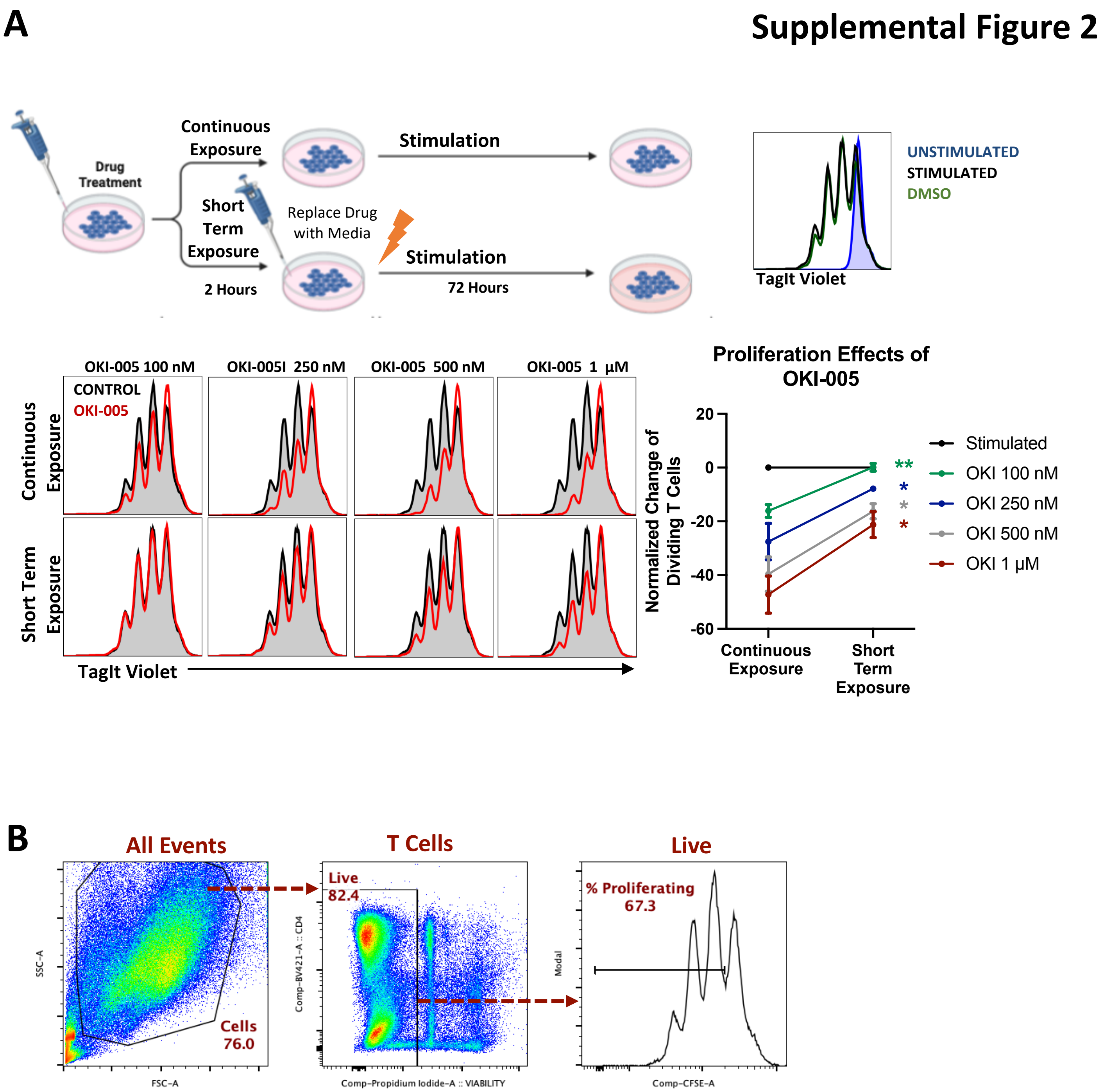

Supplemental Figure 2. OKI-005 effects on T cell proliferation are reversible. **(A)** T cells enriched from PBMCs from healthy donors were stained with TagIt Violet™ Proliferation and Cell Tracking Dye and stimulated with Dynabeads™ Human T-Activator CD3/CD28, either alone (black lines) or in the presence of the indicated concentrations of OKI-005 (red lines) for 2 hours, that was either retained (top) or removed (bottom) during stimulation, as depicted in the schematic. After 72 hours, proliferation was assessed by dilution of the proliferation dye staining. Shown are representative FACS from one of three experiments while the graph represents cumulative quantitative results of the percent proliferated after normalization with the symbol representing the mean ± SEM, n=3;* P<0.05, ** P<0.01. **(B)** Representative sequential gating schemes for evaluation of *in vitro* PBMCs from healthy donors. The initial plot in each gating scheme is gated on All events and sequential gating is indicated by arrows.

Supplemental Figure 3

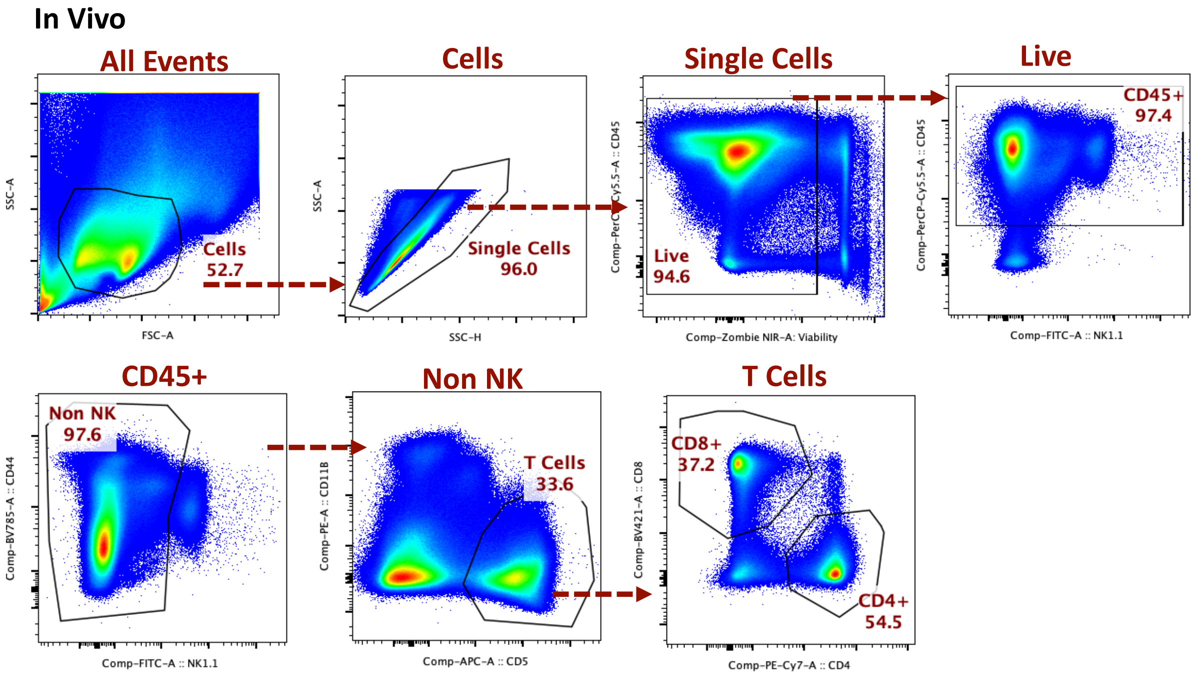

Supplemental Figure 3. Gating strategies for analyzing the immune compartment *ex vivo*. Shown are representative FACS plots of the sequential gating schemes indicated by arrows used in Figure 3 for evaluating T cells in the blood and spleen.

Supplemental Figure 4

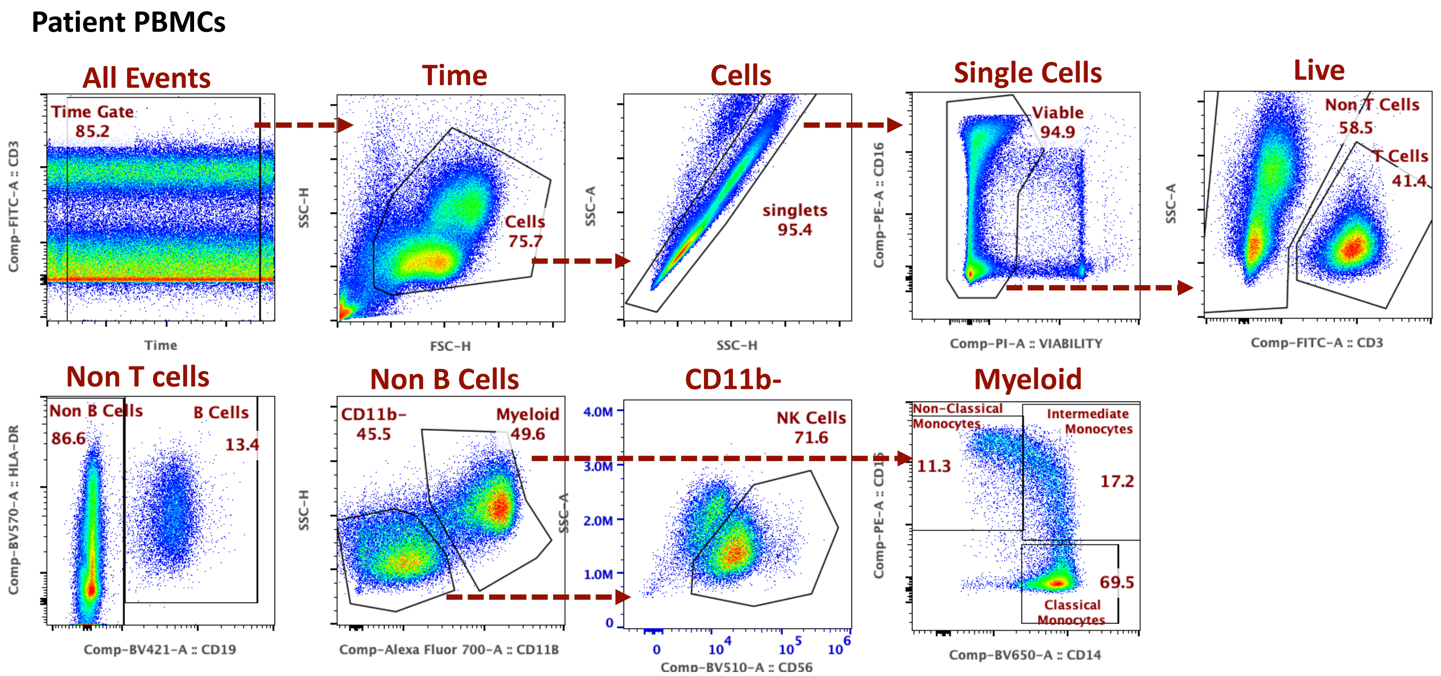

Supplemental Figure 4. Gating strategies for analyzing the immune subsets in patients. Representative sequential gating schemes indicated by arrows and titles above for quantifying the indicated immune subsets: CD4+ T Cells (CD3+ CD4+), CD8+ T Cells (CD3+ CD8+), B Cells (CD19+), NK Cells (CD56+), Transitional Monocytes (CD14+CD16+), and Classical Monocytes (CD14+CD16-) in patient PBMCs.

Supplemental Figure 5

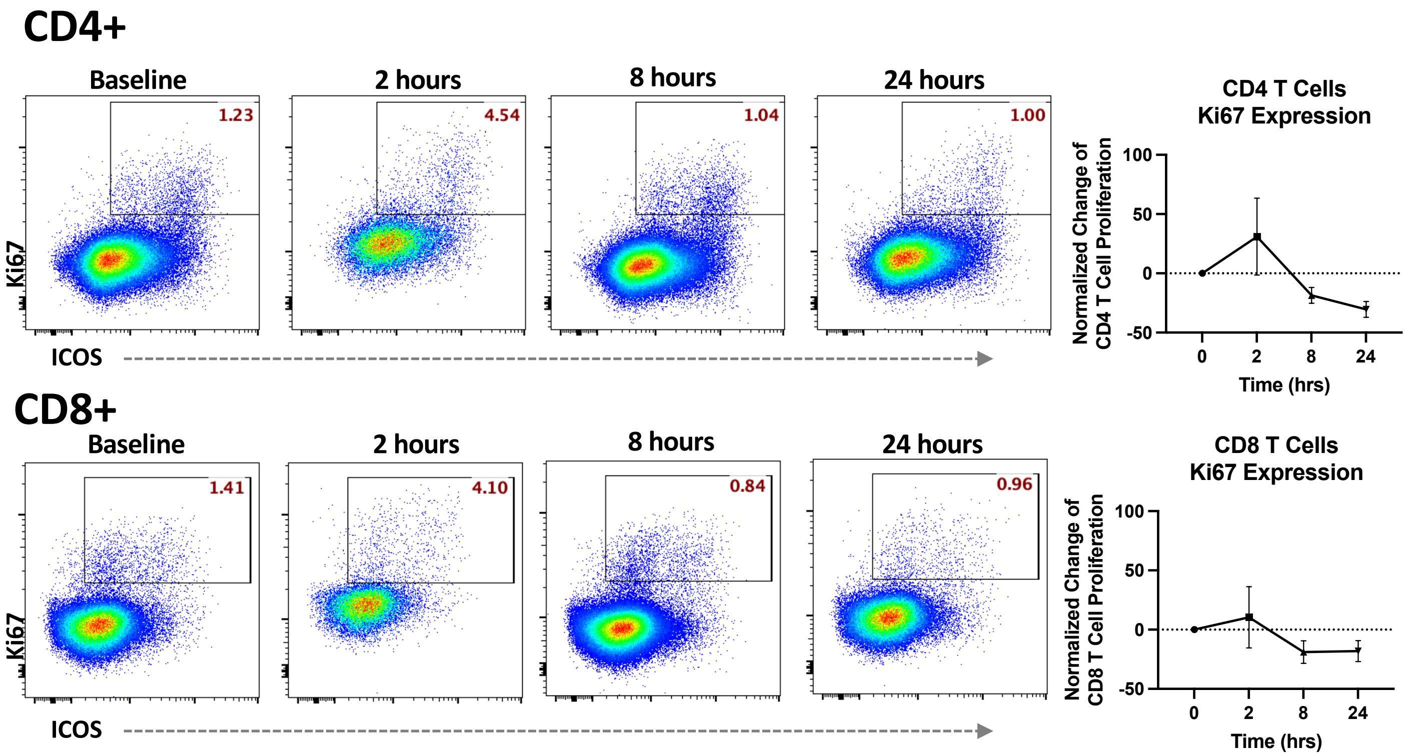

Supplemental Figure 5. Evaluation of Ki67 in T cells indicates increased frequency in patients is not due to changes in proliferation. Patient PBMCs were assessed for CD3, CD4, CD8 and intracellular Ki-67 expression following OKI-179 dosing. Flow cytometry plots are gated on CD3+CD4+ or CD3+CD8+ T cells. Graphs show cumulative data of results normalized to baseline controls within each patient for changes in frequency of Ki67+ cells normalized to baseline, with each plot representing mean ± SEM; n=10.

Supplemental Figure 6

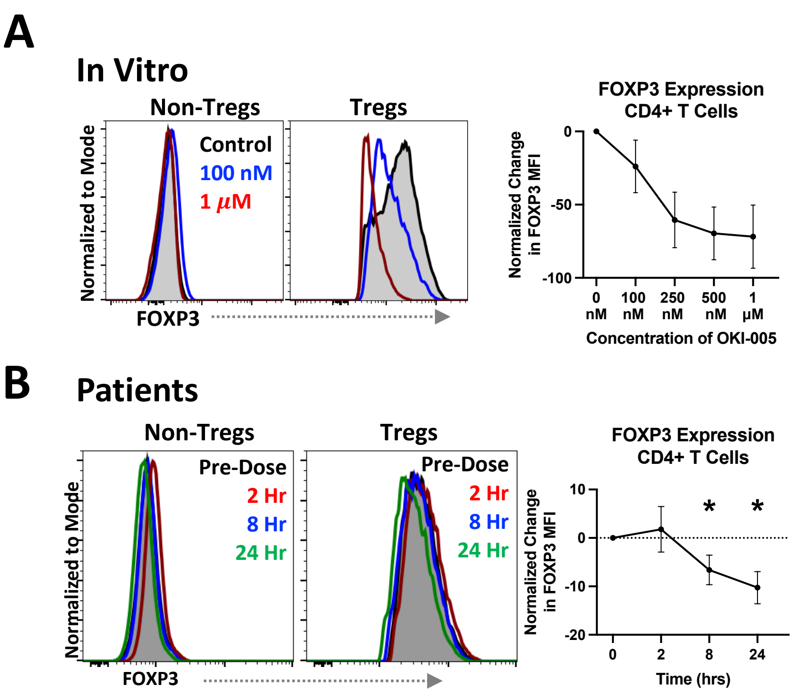

Supplemental Figure 6. OKI-005/OKI-179 affects Treg and cytotoxic T cell subsets. **(A)** PBMCs were treated with OKI-005 at doses from 0 to 1 μM for 24 hours and assessed for CD4, CD25, CD127, and FOXP3 expression. Flow cytometry plots and histograms are representative of three independent experiments. MFI of FOXP3 expression was graphed for all CD4+ cells. MFI of FOXP3 was quantified and normalized to DMSO-treated control cells; n=3; **(B)** PBMCs isolated from patients receiving OKI-179 were assessed *ex vivo* for FOXP3 expression. Representative flow cytometry plots are gated on CD3+CD4+ T cells. MFI of FOXP3 was quantified and normalized (right) to baseline sample; n=18. Results shown are cumulative with in the graphs expressed as the mean ± SEM; * $P<0.05$.

Supplemental Figure 7

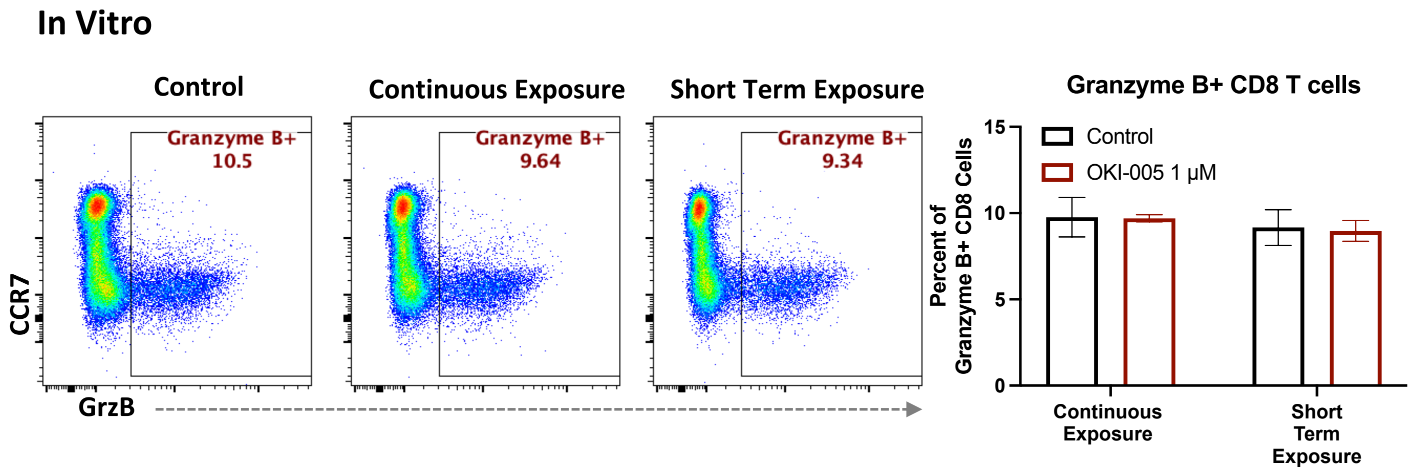

Supplemental Figure 7. OKI-005 does not alter Granzyme B expression *in vitro.* PBMCs were treated with OKI-005 at 1 μM continuously (continuous exposure), or the drug was removed after 2 hours and replaced with normal media (short term exposure). Granzyme B expression was analyzed after 8 hours by flow cytometry. Graphs show cumulative data of results with the bar representing the mean ± SEM; n=3.

Supplemental Figure 8

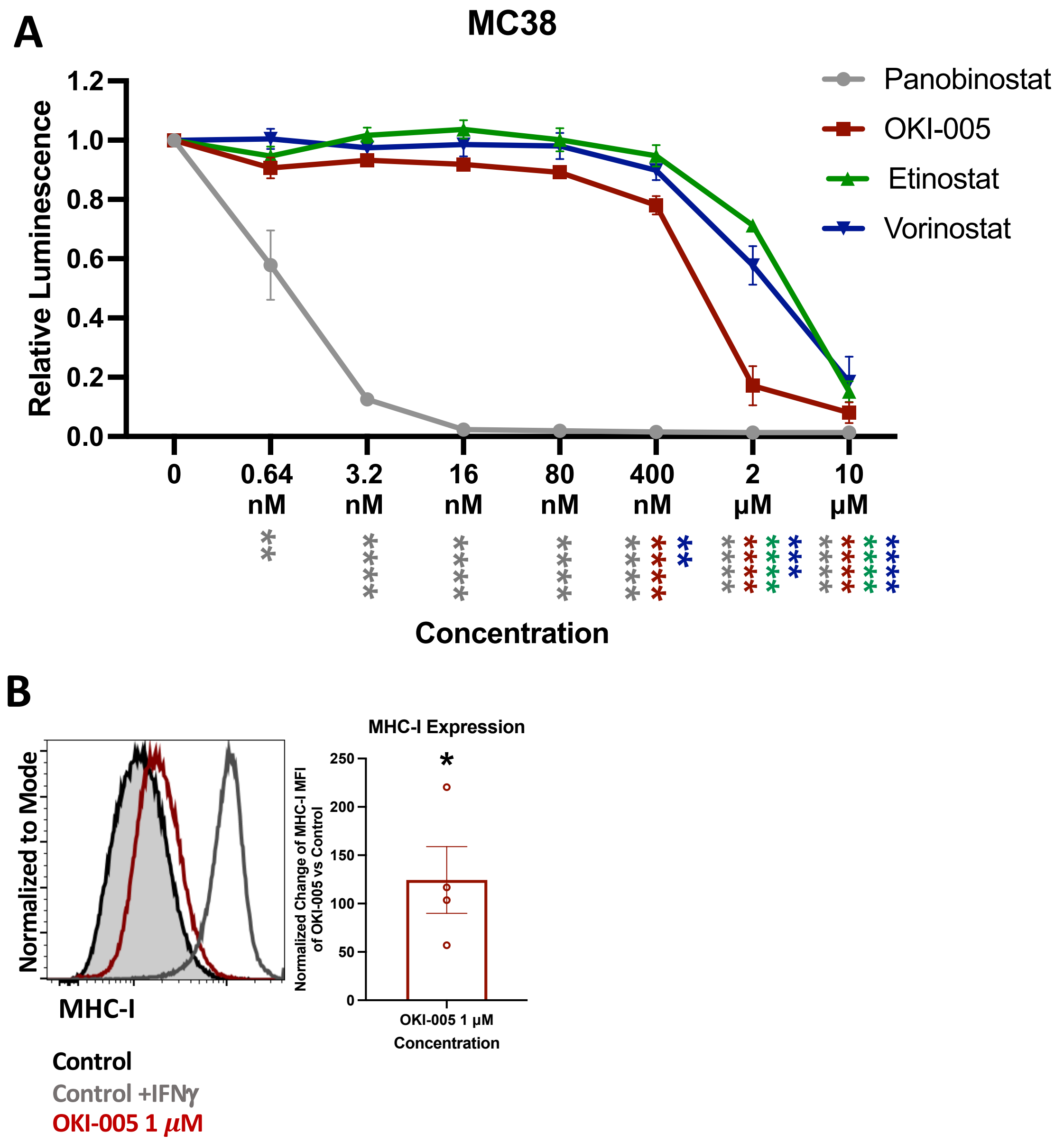

Supplemental Figure 8. OKI-005 effects on MC38 Colorectal Cancer Cells. (A) MC38 cells were treated with varying HDAC inhibitors (panobinostat, OKI-005, entinostat, and vorinostat) for 72 hours. Treatments ranging from 0.64 nM to 10 µM showed minimal direct tumor effects; n=3, in technical triplicate. Graphs show cumulative data of results normalized to DMSO controls within each experiment with each dot and error representing mean ± SEM**. (B)** MC38 cells were treated with DMSO (Negative Control), IFNγ at 50 ng/ml (Positive Control) or with OKI-005 at 1 µM for 24 hours and assessed for MHC-I expression by flow cytometry. Overlaid flow cytometry histograms show MHC-I expression of MC38 tumor cells (Left plot) and quantification using the MFI; n=4. Graphs show cumulative data of results normalized to DMSO controls within each experiment for changes in relative luminescence or MFI of MHC-I with the bar representing the mean ± SEM; * $P<0.05$, ** $P<0.01$, *** $P<0.001$, **** $P<0.0001$.

Supplemental Figure 9

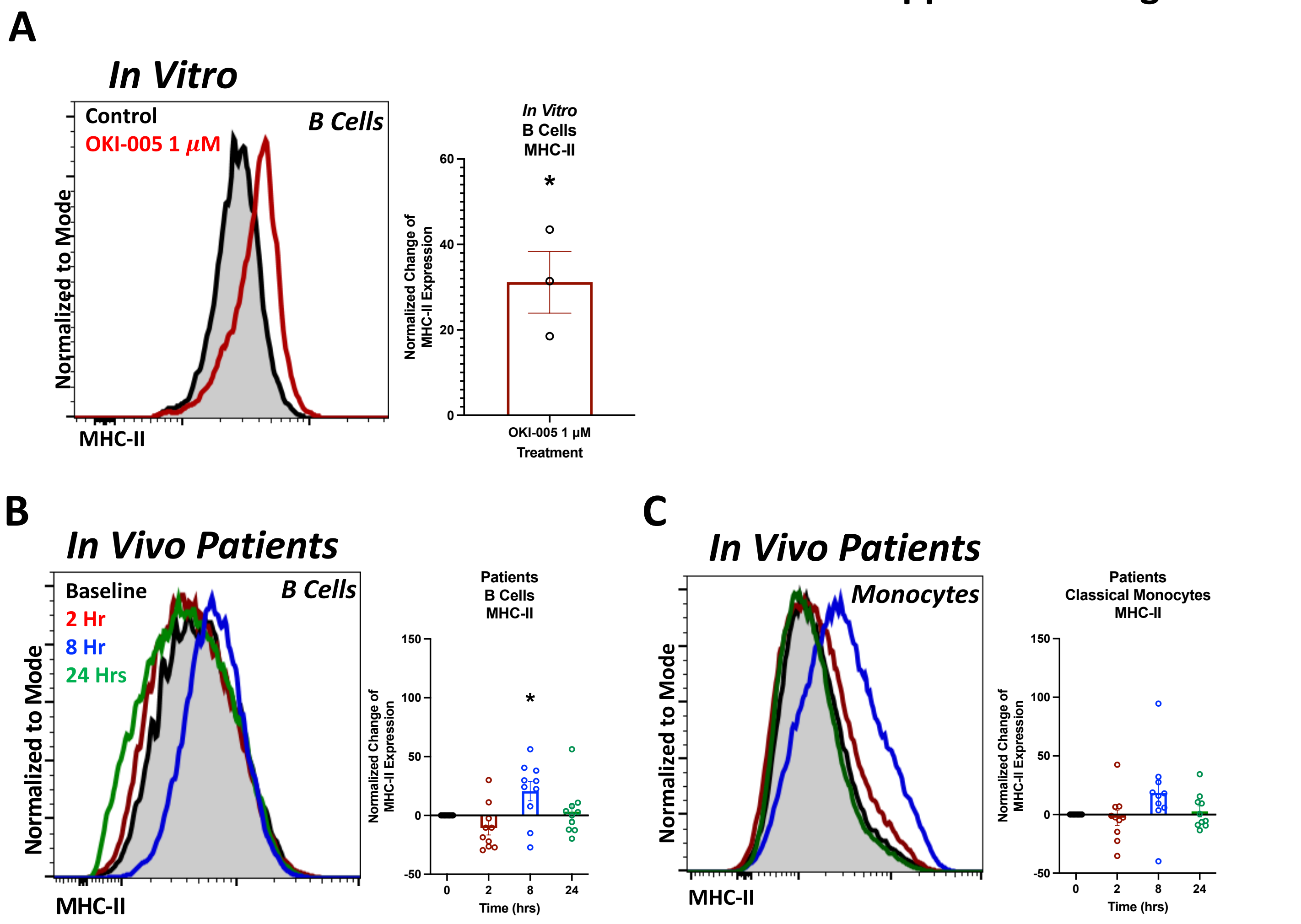

Supplemental Figure 9. Treatment with OKI-005 and OKI-179 promotes MHC-II Expression on Antigen Presenting Cells. **(A)** PBMCs were treated with 1 µM of OKI-005 for 24 hours *in vitro* and evaluated for MHC-II expression by flow cytometry analysis. Shown are representative histogram (Left) of MHC-II expression of CD19+ B cells; n=3. **(B** and **C)** PBMCs isolated from patients at timepoints indicated were stained for MHC-II expression of (**B**) CD19+ B cells or (**C**) classical monocytes at the indicated timepoints; n=10. Graphs show cumulative data of MFI of MHC-II normalized to DMSO or baseline controls within each experiment or patient with the bar representing the mean ± SEM and each symbol representing results from independent (**A**) experiments or (**B** and **C**) individuals; * P<0.05.

Supplemental Figure 10

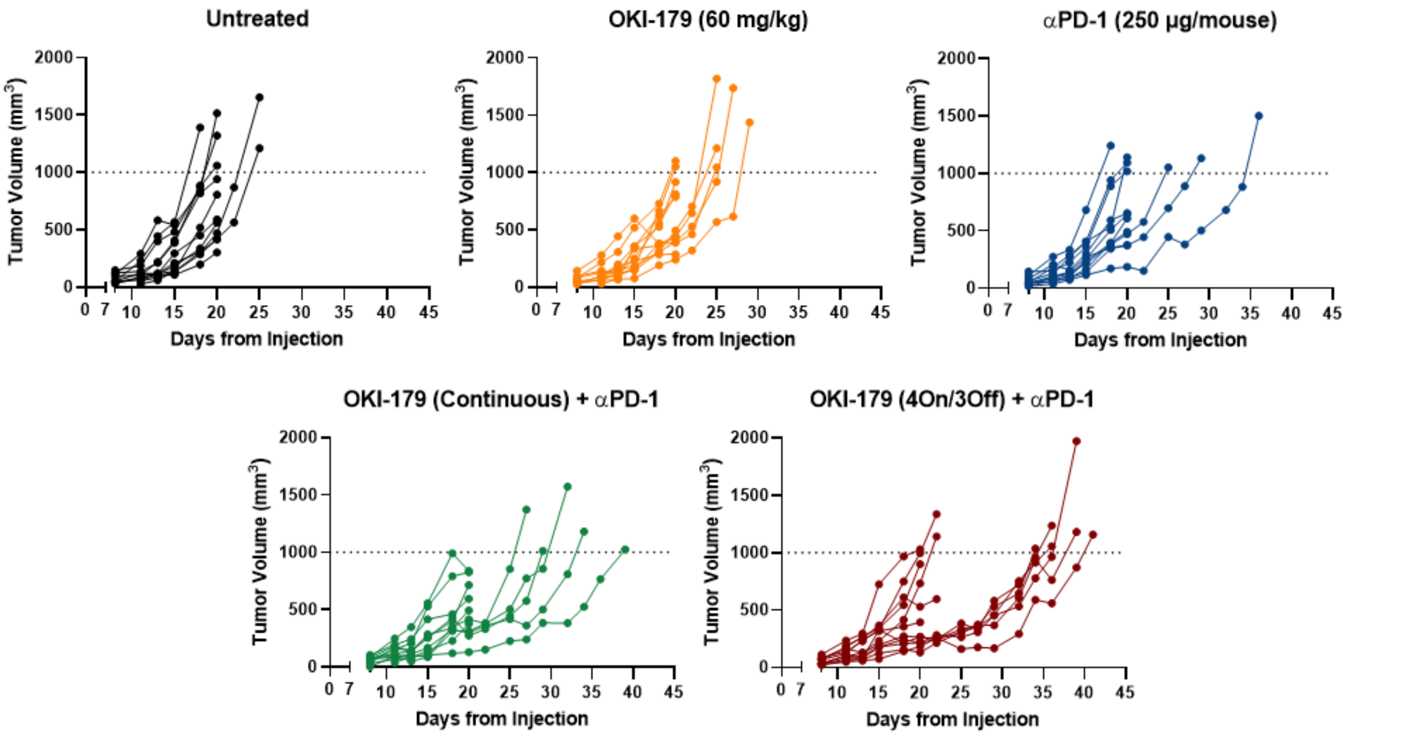

Supplemental Figure 10. Combination therapy of OKI-179 and αPD-1 slows growth of MC38 CRC Tumor growth curves of MC38 CRC tumors in Wild-Type C57Bl/6 mice that were treated with either Vehicle (Citrate Buffer), OKI-179 (60 mg/kg) alone for 30 days, αPD-1 (250 µg) three times a week (as indicated), or a combination as indicated with continuous OKI-179 (60 mg/kg) or an on/off OKI-179 dosing schedule with αPD-1 (250 µg).
